# Increased Risk of Atrial Fibrillation and Thromboembolism in Patients with Severe Psoriasis: a Nationwide Population-based Study

**DOI:** 10.1038/s41598-017-10556-y

**Published:** 2017-08-30

**Authors:** Tae-Min Rhee, Ji Hyun Lee, Eue-Keun Choi, Kyung-Do Han, HyunJung Lee, Chan Soon Park, Doyeon Hwang, So-Ryoung Lee, Woo-Hyun Lim, Si-Hyuck Kang, Myung-Jin Cha, Youngjin Cho, Il-Young Oh, Seil Oh

**Affiliations:** 1 Division of Cardiology, Department of Internal Medicine, Seoul National University Hospital, Seoul, Korea; 2 Department of Dermatology, Seoul St. Mary’s Hospital, College of Medicine, The Catholic University of Korea, Seoul, Korea; 3 Department of Biostatistics, College of Medicine, The Catholic University of Korea, Seoul, Korea; 4 Division of Cardiology, Department of Internal Medicine, Seoul National University Boramae Medical Center, Seoul, Korea; 5 Department of Cardiology, Cardiovascular Center, Seoul National University Bundang Hospital, Seongnam, Korea

## Abstract

Psoriasis increases the risk of atrial fibrillation (AF) and thromboembolic events (TE). There is limited information on the effect of psoriasis severity on AF and TE. In this study, psoriasis patients were enrolled from the Korean National Insurance Service-National Sample Cohort (2004–2008). Diagnosis and disease severity were determined from claims data. Newly diagnosed non-valvular AF and TE were identified during a 9.6-year follow-up. The effect of psoriasis severity on AF and TE was evaluated. We identified 13,385 psoriasis patients (1,947 with severe psoriasis). Severe psoriasis significantly increased the risk of AF (adjusted hazard ratio [HR_adjust_] 1.44 [95% confidence interval (CI) 1.14–1.82], p = 0.002) and TE (HR_adjust_ 1.26 [95% CI 1.07–1.47], p = 0.005); mild psoriasis did not show any significant effects. Results were similar after propensity-score matching. Risk increments of AF and TE were prominent in patients with greater cardiovascular risk. A possible limitation of our study is that it has a retrospective design, and the effect of unmeasured confounders and risk of misclassification could bias the results. To conclude, our results showed that severe, but not mild, psoriasis significantly increased AF and TE risk. AF surveillance and active stroke prevention would be beneficial in such cases.

## Introduction

Psoriasis is a complex autoimmune inflammatory skin disorder^1^. It is well known that psoriasis is associated with diabetes and hypertension, as well as other cardiovascular (CV) risk factors^2, 3^. The severe form of psoriasis further increases the risk of these comorbidities^4^. The prothrombotic tendency combined with the increased inflammatory response in severe psoriasis independently increases the risk of myocardial infarction (MI) and stroke^5^.

Recently, psoriasis has emerged as an independent risk factor for cardiac arrhythmias^6^. A strong relationship was reported between psoriasis and the incidence of atrial fibrillation (AF)^7^; increased risk of thromboembolic events (TE) has also been observed in non-valvular AF patients with psoriasis^8^. However, traditional CV risk factors, including hypertension and dyslipidemia, were not adjusted, limiting the ability to determine the effect of psoriasis on AF development^9^. Furthermore, the relationship between the severity of psoriasis and CV disease or stroke is controversial^10^.

Considering the substantial disease burden and disability rate of stroke predisposed by AF^11–13^, validating the direct effect of psoriasis on AF and related outcomes is crucial. It is important to identify subgroups particularly vulnerable to TE events and AF. We thus evaluated the influence of psoriasis on the risk of AF and TE, stratified by disease severity, in a Korean nationwide random sample cohort.

## Methods

### Data Source and Study Population

We initially recruited 1,034,777 subjects from the national sample cohort provided by the Korean National Health Insurance Service (NHIS). The cohort profile was previously described^2, 14^. In brief, the NHIS is an obligatory universal health insurance system covering approximately 97% of the population in the Republic of Korea; the remaining 3% represent the lower income population covered by the Medical Aid program. The NHIS claim database includes extensive information about demographics, medical treatments, procedures, and disease diagnoses according to the 10^th^ revised codes of International Classification of Diseases (ICD-10). The sample cohort released by the NHIS is a dynamic retrospective cohort data consisting of nationally representative random subjects, equivalent to approximately 2.2% of the entire Korean population. The database is open to any researcher whose study protocols have been approved by the official review committee. As the data were completely anonymous, the study protocol was exempt from review by the Seoul National University Hospital Institutional Review Board.

### Establishment of Study Cohort and Propensity Score Matched Cohort

**Figure 1:**
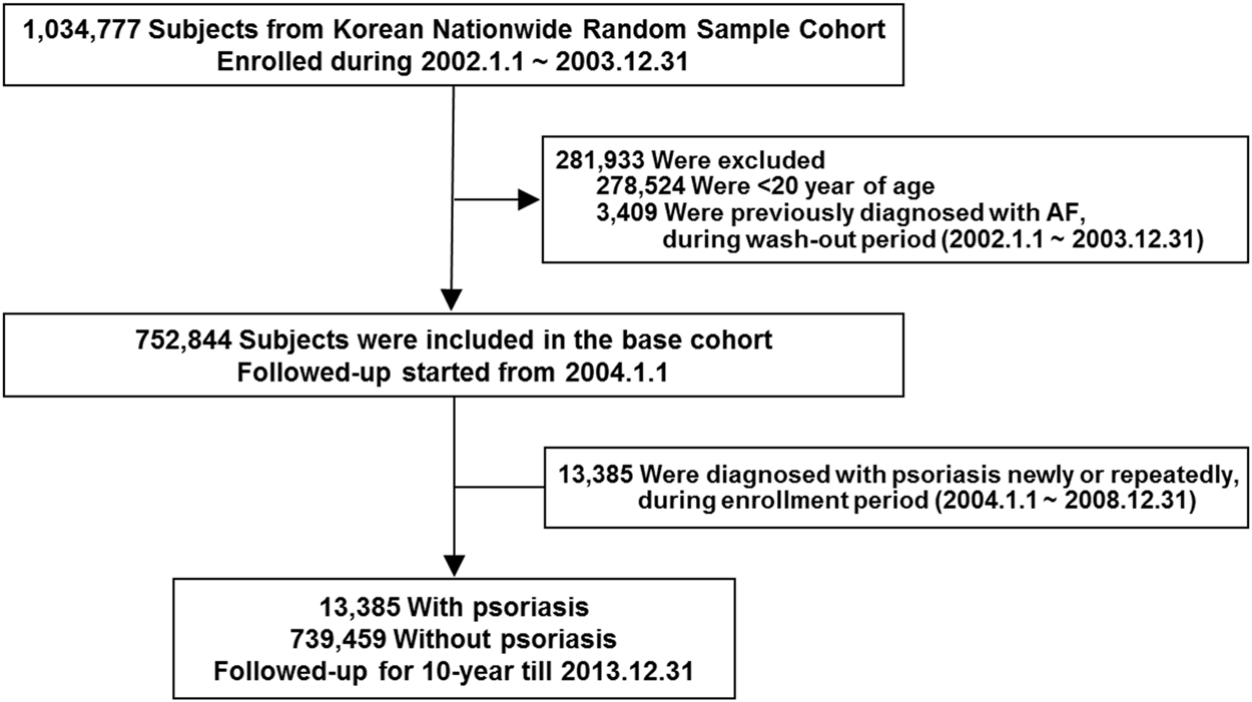
Flow of Cohort Establishment and Follow-Up. This study was a 10-year retrospective cohort study established from the Korean nationwide health insurance claim data. Abbreviations: AF, atrial fibrillation.

We excluded 278,524 subjects under the age of 20. Subsequently, 3,409 patients diagnosed with AF more than once within the first two years (from January 2002 to December 2003) after inception of the database were excluded for adequate wash-out of patients with prevalent AF (Fig. 1). Thus, 752,844 subjects were left in the base cohort and followed from January 2004. Patients newly or repeatedly diagnosed with psoriasis were identified during the next 5 years as an enrollment period. The entire cohort was divided into the psoriasis group (13,385 patients enrolled between January 2004 and December 2008) and the non-psoriasis group (739,459 participants enrolled in January 2004), and both groups were followed for a maximum of 10 years, until December 2013. Psoriasis was defined using the ICD-10 codes (L40 and M07.0-M07.3) registered by the physician(s) responsible for treatment.

Since differences in baseline characteristics could significantly affect outcomes, a propensity score matched analysis was performed to adjust the measured confounders. Variables that could potentially affect the clinical outcomes (age, gender, low income, place of residence, hypertension, diabetes mellitus, dyslipidemia, congestive heart failure, peripheral arterial disease, prior history of MI, and history of ischemic stroke) were chosen to produce propensity scores for each subject. A 1:5 matching without replacements was performed by a Greedy digit match algorithm, yielding 13,357 psoriasis patients matched with 66,785 controls. The propensity score matching processes are detailed in Supplementary Appendix.

### Defining the Severity of Psoriasis, Outcomes, and Comorbidities

Severe psoriasis was defined as psoriatic arthritis or treatment with systemic anti-psoriatic therapy, i.e. biologic drugs, cyclosporine, retinoids, or methotrexate, as recently described and validated^15–17^. The primary endpoint was the development of non-valvular AF, defined using ICD-10 codes (I48.0 – I48.4, I48.9) as described previously^2^. Secondary endpoints included the occurrence of TE, including ischemic stroke and systemic thromboembolism. AF and TE were identified by the definitions based on ICD-10 codes during the follow-up period. Comorbidities including hypertension, diabetes mellitus, dyslipidemia, congestive heart failure, peripheral arterial disease, prior history of MI, and ischemic stroke, were also defined by ICD-10 codes. The CHA_2_DS_2_-VASc score was subsequently calculated using the registered diagnostic codes. The proportion of patients who were on the platelet inhibitors (e.g. aspirin) or vitamin K antagonists (e.g. warfarin) were also identified. Definitions of outcomes, comorbidities, and the individual components of the CHA_2_DS_2_-VASc score are described in detail in Supplementary Table 1.

### Statistical Analysis

Categorical variables are presented as numbers and relative frequencies (percentages) and were compared using the Chi-squared test. Normally distributed continuous variables are expressed as mean ± standard deviation and were analyzed by using the independent sample t test. When evaluating the propensity score matched cohort, the McNemar test and the paired t test were used to compare the categorical and continuous variables, respectively. Annual event rates were described as the number of events per 1000 person-years. Hazard ratios (HR) and the corresponding 95% confidence intervals (CI) were calculated using Cox proportional hazard models. Comparison of cumulative event rates between psoriasis and non-psoriasis groups was based on Kaplan-Meier censoring estimates and performed with the log-rank test. Crude risks were analyzed in the overall cohort, and adjusted results were subsequently calculated with the multivariable Cox regression analyses. The results were validated in the propensity score matched cohort. Subgroup analyses divided by multiple CV risk factors were subsequently performed. All p-values were two-sided, and a value less than 0.05 was considered statistically significant. Statistical analyses were performed using SAS version 9.3 (SAS Institute, Cary, NC, USA), Stata statistical software release 12 (StataCorp, College Station, TX, USA), and SPSS statistical package version 19.0 (SPSS Inc., Chicago, IL, USA).

## Results

### Baseline Characteristics of the Overall Cohort

Table 1 reports baseline characteristics and demographic features of the severe psoriasis, mild psoriasis, and non-psoriasis groups. The number of patients with severe psoriasis was 1,947, representing 14.5% of all psoriasis patients. The overall CV risk status was highest in the severe psoriasis group, followed by the mild psoriasis group. The severe psoriasis group was composed of older patients, with a greater proportion of males, and showed a higher proportion of well-known CV risk factors, including hypertension, diabetes, and dyslipidemia, as well as previous history of MI and ischemic stroke. The proportion of patients who were on platelet inhibitors and vitamin K antagonists was also significantly higher in the severe psoriasis group than the mild psoriasis group or non-psoriasis group. The mean duration of follow-up was shorter in the psoriasis group (severe, 9.46 years with 17,786 patient-years; mild, 9.55 years with 103,764 patient-years) than in the non-psoriasis group (9.60 years with 7,339,935 observational patient-years).

**Table 1:** Baseline Characteristics of Study Patients.

Characteristics	Non-psoriasis (N = 739,459)	Mild psoriasis (N = 11,438)	Severe psoriasis (N = 1,947)	P value
Age (year)	43.5 ± 15.6	45.7 ± 15.8	53.1 ± 14.6	<0.001
20-39	337,849 (45.7%)	4,536 (39.7%)	395 (20.3%)	
40-64	313,241 (42.4%)	5,197 (45.4%)	1,053 (54.1%)	
≥65	88,369 (12.0%)	1,705 (14.9%)	499 (25.6%)	
Male	362,755 (49.1%)	5,979 (52.3%)	729 (37.4%)	<0.001
Low income^*^	124,260 (16.8%)	1,498 (13.1%)	294 (15.1%)	<0.001
Rural residents	387,939 (52.5%)	5,956 (52.1%)	1,225 (62.9%)	<0.001
Hypertension	79,687 (10.8%)	2,029 (17.7%)	530 (27.2%)	<0.001
Diabetes mellitus	26,863 (3.6%)	171 (8.8%)	762 (6.7%)	<0.001
Dyslipidemia	46,719 (6.3%)	1,315 (11.5%)	325 (16.7%)	<0.001
Congestive heart failure	9,259 (1.3%)	282 (2.5%)	70 (3.6%)	<0.001
Peripheral arterial diseases	8,814 (1.2%)	272 (2.4%)	90 (4.6%)	<0.001
History of myocardial infarction	7,108 (1.0%)	193 (1.7%)	26 (1.3%)	<0.001
History of ischemic stroke	7,277 (1.0%)	236 (2.1%)	52 (2.7%)	<0.001
CHA_2_DS_2_-VASc score				<0.001
0 or 1	627,504 (84.9%)	9,120 (79.7%)	1,224 (62.9%)	
≥ 2	111,955 (15.1%)	2,318 (20.3%)	723 (37.1%)	
Use of platelet inhibitors	33,133 (4.5%)	787 (6.9%)	201 (10.3%)	<0.001
Use of vitamin K antagonists	882 (0.1%)	23 (0.2%)	3 (0.2%)	0.040
Duration of follow-up (year)	9.60 ± 1.42	9.55 ± 1.47	9.46 ± 1.62	<0.001
Number of patient-years	7,339,935	103,764	17,786	—

^*^Denotes subjects with annual income lower than 20% among total population.

### Incidence Rates and Relative Risks of Clinical Outcomes According to Psoriasis Severity

**Figure 2:**
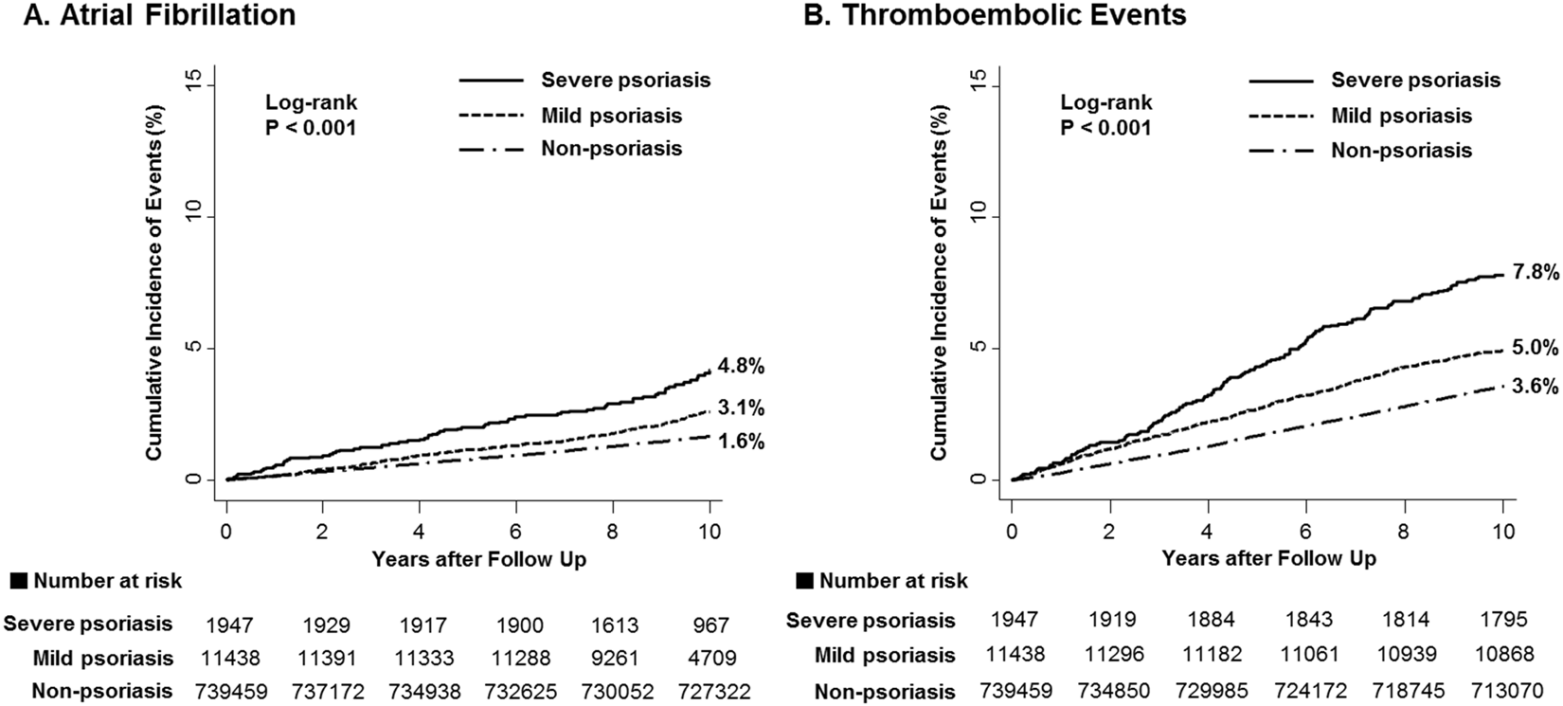
Comparison of Cumulative Incidence of Atrial Fibrillation and Thromboembolic Events According to the Severity of Psoriasis. Kaplan-Meier curves with cumulative hazards of atrial fibrillation **(A)** and thromboembolic events **(B)** compared by severity of psoriasis are presented. The adjusted hazard ratios were calculated by multivariable Cox regression models, including age over 65, gender, income level, resident area, hypertension, diabetes, dyslipidemia, congestive heart failure, peripheral arterial disease, prior history of stroke and myocardial infarction as covariates. Abbreviations: CI, confidence interval; HR, hazard ratio; N/A, non-applicable.

The psoriasis group had higher incidences of AF and TE compared to the non-psoriasis group. Table 2 reports the number of events, calculated incidence rates, and unadjusted and adjusted HRs for AF and TE according to psoriasis severity. Patients with severe psoriasis had more clinical events than those with mild psoriasis (incidence rates of AF, 4.05 vs. 2.48; TE, 8.14 vs. 5.13 per 1,000 person-years in the severe and mild psoriasis groups, respectively). The crude HRs of AF and TE were significantly higher in both the severe and mild psoriasis groups. However, after adjusting for age, gender, income level, area of residence, hypertension, diabetes, dyslipidemia, congestive heart failure, peripheral arterial disease, prior history of ischemic stroke or MI, and use of platelet inhibitors or vitamin K antagonists, only severe psoriasis remained an independent predictor of AF (HR_adjust_ 1.44 [1.14–1.82], p = 0.002) and TE (HR_adjust_ 1.26 [1.07–1.47], p = 0.005), whereas mild psoriasis did not (for AF, HR_adjust_ 1.10 [0.97–1.25], p = 0.133; for TE, HR_adjust_ 1.04 [0.96–1.13], p = 0.382). Cumulative hazard curves for AF and TE according to psoriasis severity are presented in Fig. 2.

**Table 2:** Risk of the Atrial Fibrillation and Thromboembolic Events in Psoriasis Patients by the Severity.

Outcomes	No. of events	Incidence^*^	Unadjusted		Multivariable adjusted^†^		PS-matched	
			HR (95% CI)	P value	HR (95% CI)	P value	HR (95% CI)	P value
**Atrial Fibrillation**								
Non-psoriasis	12143/739459	1.65	1.00 (reference)	—	1.00 (reference)	—	1.00 (reference)	—
Mild psoriasis	257/11438	2.48	1.51 (1.34–1.71)	<0.001	1.10 (0.97–1.24)	0.133	1.06 (0.93–1.22)	0.365
Severe psoriasis^‡^	72/1947	4.05	2.47 (1.96–3.11)	<0.001	1.44 (1.14–1.82)	0.002	1.77 (1.39–2.24)	<0.001
**Thromboembolic Events**								
Non-psoriasis	26406/739459	3.63	1.00 (reference)	—	1.00 (reference)	—	1.00 (reference)	—
Mild psoriasis	571/11438	5.13	1.41 (1.30–1.54)	<0.001	1.04 (0.96–1.13)	0.382	0.98 (0.90–1.08)	0.707
Severe psoriasis	152/1947	8.14	2.25 (1.91–2.63)	<0.001	1.26 (1.07–1.47)	0.005	1.55 (1.32–1.84)	<0.001

^*^Incidence rates were calculated per 1000 patient-years, within the population who were over 20 years old and not previously diagnosed with atrial fibrillation.

^†^Multivariable adjusted hazard ratios were calculated by Cox regression models, including age over 65, gender, income level, resident area, hypertension, diabetes, dyslipidemia, congestive heart failure, peripheral arterial disease, prior history of stroke, prior history of myocardial infarction, use of platelet inhibitors, and use of vitamin K antagonists as covariates.

^‡^Severe psoriasis patients included those with psoriatic arthritis, and those receiving systemic anti-psoriatic treatment, as previously established and validated.

Abbreviations: CI, confidence interval; HR, hazard ratio; PS, propensity score.

### Propensity Score Matched Analysis of AF and TE Risk by Psoriasis Severity

All observable clinical characteristics and antithrombotic medication status became equivalent between the psoriasis and non-psoriasis groups after propensity score matching, with the exception of history of MI (Supplementary Table 2). Success of propensity score matching was assessed by calculating standardized differences in the baseline characteristics (Supplementary Figure 1). Differences for all matched covariates were less than 10%, supporting the assumption of sufficient balance achievement between patients and controls.

Consistent with results from multivariable adjusted analysis, only severe psoriasis was associated with an increased risk of AF (HR 1.77 [1.39–2.24], p < 0.001) and TE (HR 1.55 [1.32–1.84], p < 0.001), while mild psoriasis was not (HR 1.06 [0.93–1.22], p = 0.365 and HR 0.98 [0.90–1.08], p = 0.707, respectively). Supplementary Figure 2 reports cumulative hazard curves from the propensity score matched cohort.

### Subgroup Analyses

**Figure 3:**
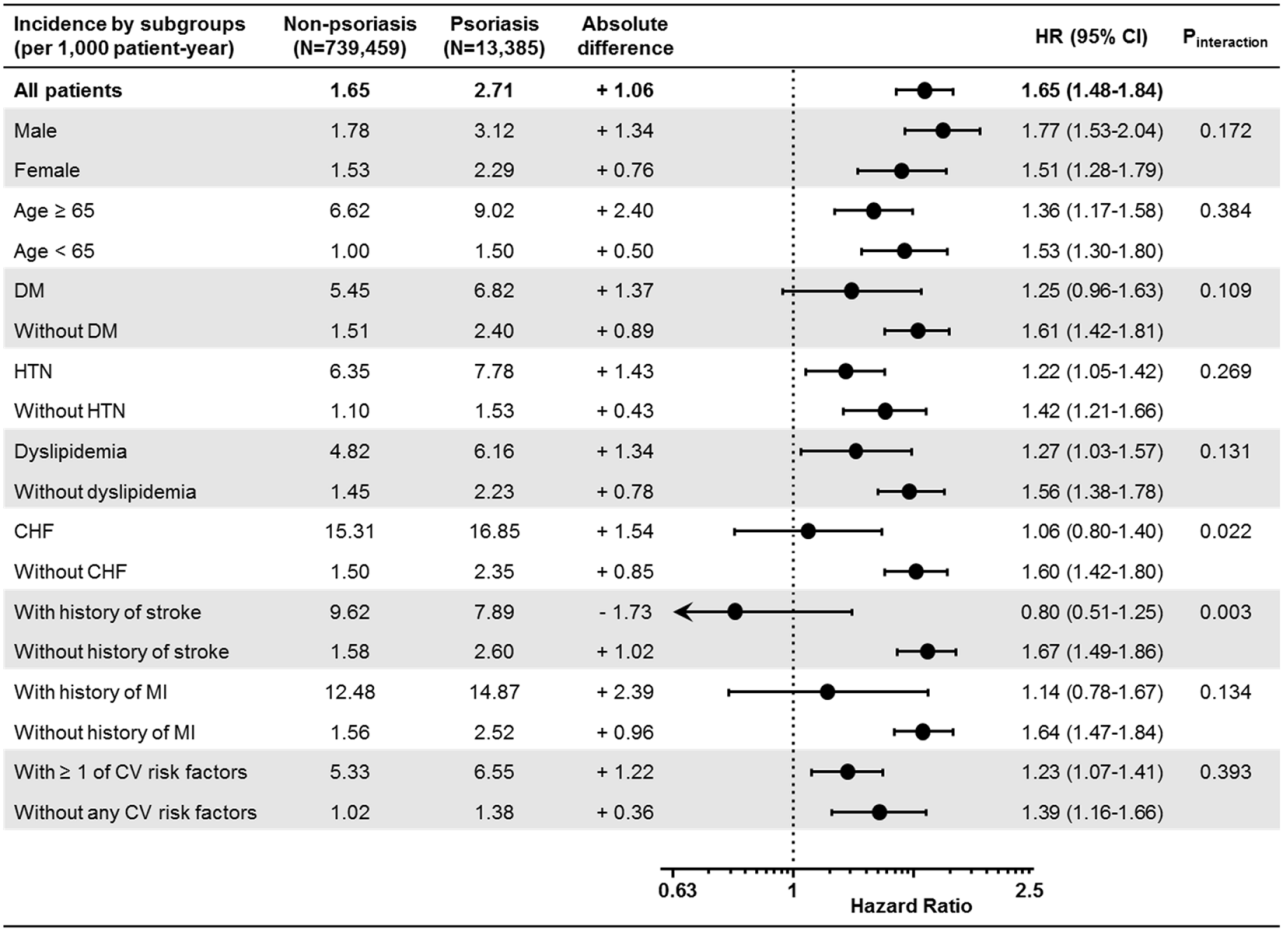
Subgroup Analyses for Atrial Fibrillation Risk in Psoriasis Patients. The effects of psoriasis on the risk of AF were shown to be weaker in subgroups with well-known CV risk factors than those without. However, the absolute increases of incidence rates were still much higher in risky subgroups. Abbreviations: CHF, congestive heart failure; CI, confidence interval; cardiovascular, cardiovascular; DM, diabetes mellitus; HR, hazard ratio; HTN, hypertension; MI, myocardial infarction.

Subgroup analysis in psoriasis patients showed a consistently higher risk of AF and TE in most of the exploratory subgroups (Fig. 3 and Supplementary Figure 3). For AF, significant interactions were observed in the subgroups of patients with congestive heart failure (P_interaction_ = 0.002) and history of stroke (P_interaction_ = 0.003). For TE, in contrast, significant interactions were found in subgroups based on age over 65 (P_interaction_ = 0.007) and diabetes (P_interaction_ = 0.002). In both subgroup analyses, the effect of psoriasis on AF and TE was relatively weaker in subgroups with well-known CV risk factors than in those without. However, in the viewpoint of absolute risk increments, the incidence rates of AF and TE were much higher in those with traditional CV risk factors.

## Discussion

In this Korean nationwide cohort established from the national health insurance database, the effect of psoriasis on the risk of AF and TE was primarily observed in patients with severe disease, even after thorough adjustments. Though the relative risk effect of psoriasis appeared to be smaller in low CV risk groups, the absolute increase in the incidence rates associated with psoriasis was still greater in patients with CV risk factors than in those without.

There has been controversy concerning the effect of mild psoriasis on CV outcomes^9, 10, 18, 19^. With the sufficiently large sample size and comprehensive adjustment in the present study, we conclude that mild psoriasis actually has no influence on the occurrence of AF or TE.

To date, the consensus regarding the effect of psoriasis on AF and TE has been established by the Danish nationwide population-based study conducted by Ahlehoff *et al*.^7^. The authors concluded that both mild and severe psoriasis significantly increased the risk of AF and stroke. However, several studies did not find a strong association between mild psoriasis and CV or stroke^4, 8, 20, 21^. The results comparing these studies are summarized in Supplementary Table 3. Considering the lower AF incidence rates in our study than in studies performed in the United States or Denmark, racial difference could have influenced the results^22, 23^. In addition, although the Danish cohort study adjusted for several comorbidities, some important CV risk factors were not adjusted in the multivariable analysis, which might overestimate the effect of psoriasis severity on AF. The various definitions of severe psoriasis across studies further limit the confidence in previous conclusions. Concerns about misclassification bias have been evoked, and recently, the definition of severity was revised using the claims for systemic anti-psoriatic medications^15^. This new definition was subsequently validated in an external cohort^16^.

As a well-established nationwide large cohort study, the present analysis used the most updated and sophisticated severity definitions, included very high risk patients with previous MI or ischemic stroke who were excluded from previous studies, and adjusted for all available covariates including most CV risk factors and prescription status of antithrombotic medication. We thus found reliable evidence indicating that severe psoriasis is an independent predictor of AF and TE, while mild psoriasis is not.

Active inflammation underlies the pathophysiology of both severe psoriasis and AF. Chronic inflammation is also a well-known catalyst of the left atrial remodeling process^24, 25^, further accelerating the occurrence of AF, which is supported by histological evidence of inflammatory infiltrates and oxidative damage within atrial tissue taken from AF patients^26, 27^.

Though several studies have reported that stroke is strongly associated with psoriasis disease activity^4, 7, 20, 21^, the exact mechanism remains unclear. The systemic inflammation caused by severe psoriasis could also affect the cardiovascular risk^28^. By proving a strong relationship between severe psoriasis and AF as well as TE, this study provides the epidemiological evidence that might explain the missing link between psoriasis and stroke.

In the subgroup analyses, the relative risk effects of psoriasis were consistently smaller in high CV risk patients. Powerful CV risk factors, such as prior history of stroke or MI, showed significant interactions. Samarasekera *et al*.^9^ reported similar findings in their meta-analysis. Although higher relative risk of CV was also observed in younger patients, the absolute risk was far greater in older individuals, suggesting that the numerically high relative risk found in younger patients should be interpreted carefully. In the same manner, despite the high hazard ratios reflecting an increased relative risk in the lower CV risk group, the effect of psoriasis assessed by absolute incidence increment was actually trivial. Conversely, a large increase in absolute risk was observed in the higher CV risk group, which should not be overlooked due to the numerically lower relative risk.

Though the prevalence of psoriasis varies from 1% to 8.5% according to ethnicity or latitude of residence^29, 30^, Tsai TF *et al*.^31^ reported that approximately 3% of Asian adults have moderate to severe psoriasis. However, since the majority of psoriasis patients initially visit dermatologists for their skin manifestations, appropriate management to reduce the risk of AF and TE is difficult to achieve in clinical practice. Therefore, physicians who primarily examine and treat psoriasis patients should understand and inform patients of the increased risk of AF and TE with severe disease. Considering the higher absolute risk increment with psoriasis among higher CV risk patients, it is also necessary to regularly monitor the development of traditional CV risk factors in psoriasis patients.

Several limitations should be discussed. First, despite being a large cohort study with high statistical power, this study has the innate limitations of a retrospective, observational design, making it impossible to clearly determine the causal relationship between psoriasis and AF or TE. Second, we did not adjust for the effect of unmeasured confounders, e.g. obesity, waist circumference, familial history of CV, obstructive sleep apnea, alcohol intake, and medications other than antithrombotic agents. Notably, it was impossible to adjust for the effect of medications on outcome variables. However, since we performed thorough multivariable adjustments and PS-matching procedures as much as possible, the effect of different baseline characteristics and medication status might be minimal. Third, because we used only diagnostic codes in claims data without laboratory results, our data might be skewed due to misclassification or underestimation bias. Nevertheless, the methodology used in this study has recently been validated in various studies^2, 32–34^. Finally, the lack of data regarding the time point of first psoriasis diagnosis also made it impossible to adjust the time-varying effect associated with exposure to psoriasis.

## Conclusions

We found that severe psoriasis was strongly associated with increased risk of AF and TE, whereas mild psoriasis was not. The relative risk effect of psoriasis in patients with CV risk factors seemed to be underestimated, with the absolute risk increment being larger in high risk groups.

### Data availability statement

The datasets generated during and/or analysed during the current study are available from the corresponding author on reasonable request.

As the data were completely anonymous, the study protocol was exempt from review by the Seoul National University Hospital Institutional Review Board. (IRB No. E-1607-103-776).

## Electronic supplementary material

Supplementary Information
