# Supplementary material for: Increased Risk of Atrial Fibrillation and Thromboembolism in Patients with Severe Psoriasis: a Nationwide Population-based Study
Source: Sci Rep. 2017 Aug 30;7:9973. doi: 10.1038/s41598-017-10556-y (PMC5577288; doi:10.1038/s41598-017-10556-y)
Supplement: Supplementary file 1 — Supplementary Information [file 41598_2017_10556_MOESM1_ESM.pdf]

Supplementary Appendix

Increased Risk of Atrial Fibrillation and Thromboembolism in Patients with Severe Psoriasis: a Nationwide Population-based Study

Tae-Min Rhee, MD[1], Ji Hyun Lee, MD[2], Eue-Keun Choi, MD, PhD[1,*], Kyung-Do Han, PhD[3], HyunJung Lee, MD[1], Chan Soon Park, MD[1], Doyeon Hwang, MD[1], So-Ryoung Lee, MD[1], Woo-Hyun Lim, MD[4], Si-Hyuck Kang, MD[5], Myung-Jin Cha, MD[1], Youngjin Cho, MD[5], Il-Young Oh, MD, PhD[5], Seil Oh, MD, PhD, FHRS[1]

[1]Division of Cardiology, Department of Internal Medicine, Seoul National University Hospital, Seoul, Korea; [2]Department of Dermatology, Seoul St. Mary's Hospital, College of Medicine, The Catholic University of Korea, Seoul, Korea; [3]Department of Biostatistics, College of Medicine, The Catholic University of Korea, Seoul, Korea; [4]Division of Cardiology, Department of Internal Medicine, Seoul National University Boramae Medical Center, Seoul, Korea; [5]Department of Cardiology, Cardiovascular Center, Seoul National University Bundang Hospital, Seongnam, Korea

Contents of the Supplementary Appendix

I. Supplementary Methods

II. Supplementary Tables

III. Supplementary Figures

IV. Supplementary Figure Legends

V. References

I. Supplementary Methods

Establishment of Propensity Score Matched Cohort

Since differences in baseline characteristics could significantly affect the outcomes, a propensity score matched analysis was performed to adjust the measured confounders. A multivariable logistic regression model was used to generate propensity scores which indicate the probability that one would have psoriasis. The covariates used to calculate the propensity scores were listed as follows: age, gender, low income, resident place (urban or rural), hypertension, diabetes mellitus, dyslipidemia, congestive heart failure, peripheral arterial disease, prior history of MI, and history of ischemic stroke. As the missing values were absent across all the covariates, it was possible to calculate propensity scores completely for all of 752,844 subjects. A 1:5 matching process without replacements was performed by a greedy algorithm with a caliper width of 0.6 standard deviations, yielding 13,357 psoriasis patients matched with 66,785 controls.

Defining the Severity of Psoriasis and Comorbidities

Severe psoriasis patients were identified as those with psoriatic arthritis or those receiving systemic antipsoriatic treatment, i.e. treatment with biologic drugs (ATC codes L04AB01,

L04AB02, L04AB04, L04AC05, L04AA21), cyclosporine (ATC code L04AD01), retinoids (ATC code D05BB), or methotrexate (ATC codes L03BA01 and L04AX03), as recently described and validated.[1-3]

Comorbidities including hypertension, diabetes mellitus, dyslipidemia, congestive heart failure, peripheral arterial disease, prior history of MI and ischemic stroke, were also defined by ICD-10 codes. To minimize the underestimation or misclassification, we used a 1-year period for detecting comorbidities, except for congestive heart failure and history of MI using 3-year period. The comparison of the prevalence of comorbidities extracted by abovementioned definitions versus previously reported in external cohort studies from the Korean National Health and Nutrition Examination Survey[4, 5] showed similar results, ensuring the validity of definition by ICD-10 codes. The CHA_2DS_2-VASc score was subsequently calculated using the registered diagnostic codes, 1-year prior to the initial diagnosis of AF. The proportion of patients who were on the platelet inhibitors (e.g. aspirin) or vitamin K antagonists (e.g. warfarin) were also identified. Definitions of outcomes, comorbidities and the individual components of CHA_2DS_2-VASc score are described in detail in the **Supplementary Table 1**.

II. Supplementary Tables

Supplementary Table 1. List of the definitions of outcomes, comorbidities and the individual components of CHA_2DS_2-VASc score.

	ICD-10 codes	Additional definitions
**Comorbidities**		
Hypertension	I10-I15	Hospitalization ≥ 1 or Outpatient visit ≥ 2
Type 2 DM	E11-E14	Hospitalization ≥ 1 or Outpatient visit ≥ 2<br>With additional claims for the oral antidiabetic agents or insulin
Dyslipidemia	E78	Any of hospitalization or outpatient visit ≥ 1
CHF	I50	Any of hospitalization or outpatient visit ≥ 1
PAD	I70, I73	Hospitalization ≥ 1 or Outpatient visit ≥ 2
Prior MI	I21, I22	Any of hospitalization or outpatient visit ≥ 1
Prior ischemic stroke	I63, I64	Hospitalization ≥ 1 or Outpatient visit ≥ 2
**CHA_2DS_2-VASc score**		
CHF	I50	Any of hospitalization or outpatient visit ≥ 1
Hypertension	I10-I15	Hospitalization ≥ 1 or Outpatient visit ≥ 2
DM	E11-E14	Hospitalization ≥ 1 or Outpatient visit ≥ 2<br>With additional claims for the oral antidiabetic agents or insulin
Stroke	I63, I64	Hospitalization ≥ 1 or Outpatient visit ≥ 2
TIA	G45.8, G45.9	Any of hospitalization or outpatient visit ≥ 1
Systemic TE	I26, I74	Any of hospitalization or outpatient visit ≥ 1
Vascular disease		
Prior MI	I21, I22	Any of hospitalization or outpatient visit ≥ 1
PAD	I70, I73	Hospitalization ≥ 1 or Outpatient visit ≥ 2
Aortic plaque	I70.0	Hospitalization ≥ 1 or Outpatient visit ≥ 2
**Outcomes**		
Nonvalvular AF	I48.0-I48.4, I48.9	Hospitalization ≥ 1 or Outpatient visit ≥ 2<br>With excluding mitral stenosis (I05.0, I05.2, I05.9) and mechanical heart valves (Z95.2-Z95.4)
Ischemic stroke	I63, I64	Any hospitalization ≥ 1<br>With additional claims for the imaging studies (brain CT or MRI)
Systemic TE	I26, I74	Any of hospitalization or outpatient visit ≥ 1

Abbreviations: AF, atrial fibrillation; CHF, congestive heart failure; CT, computed tomography; DM, diabetes mellitus; ICD-10, 10th revision of international classification of diseases; MI, myocardial infarction; MRI, magnetic resonance imaging; PAD, peripheral arterial disease; TE, thromboembolism; TIA, transient ischemic attack.

Supplementary Table 2. Baseline Characteristics of Study Patients from Propensity Score Matched Cohort.

Characteristics	Matched control* (N=66,785)	Psoriasis (N=13,357)	P value for matched comparison
Age (year)	46.8 ± 15.8	46.8 ± 15.8	0.913
20-39	24,570 (36.8%)	4,931 (36.8%)	
40-64	31,412 (47.0%)	6,244 (46.7%)	
≥65	10,803 (16.2%)	2,182 (16.3%)	
Male	33,447 (50.1%)	6,695 (50.1%)	0.930
Low income†	8,733 (13.1%)	1,781 (13.3%)	0.421
Rural residents	35,846 (53.7%)	7,163 (53.6%)	0.922
Hypertension	12,864 (19.3%)	2,537 (19.0%)	0.473
Diabetes	4,491 (6.7%)	924 (6.9%)	0.417
Dyslipidemia	8,150 (12.2%)	1,625 (12.2%)	0.904
Congestive heart failure	1,581 (2.4%)	335 (2.5%)	0.331
Peripheral arterial diseases	1,642 (2.5%)	360 (2.7%)	0.110
History of myocardial infarction	909 (1.4%)	214 (1.6%)	0.034
History of ischemic stroke	1,245 (1.9%)	270 (2.0%)	0.223
CHA_2DS_2-VASc score			0.159
0 or 1	52,085 (78.0%)	10,343 (77.4%)	
≥2	14,700 (22.0%)	3,014 (22.6%)	
Use of platelet inhibitors	4,884 (7.3%)	972 (7.3%)	0.884
Use of vitamin K antagonists	145 (0.2%)	26 (0.2%)	0.608

Severe psoriasis	0 (0.0%)	1,941 (14.5%)	-
Duration of follow-up (year)	9.52 ± 1.57	9.54 ± 1.49	0.139
Number of patient-years	660,615	121,304	-

[*] Matched control group was extracted by 1:5 propensity score matching algorithm.

[†] Denotes subjects with annual income lower than 20% among total population.

Supplementary Table 3. Summary of Previous Evidence Regarding Risk of AF and TE According to the Severity of Psoriasis

Study	Design	Total Study Population (N)	Patients (N)	Follow-Up Duration (Y)	Incidence of Events*	Risk of Events
**Atrial Fibrillation**						
Ahlehoff et al. (2012)	Retrospective cohort	4,518,484	Mild : 36,765	5.0	4.67	RR 1.22 (1.14-1.30)
	(Denmark)		Severe : 2,793	4.7	5.96	RR 1.53 (1.23-1.91)
Armstrong et al. (2013)	Retrospective cohort	8,312	Mild : 1,773	4.3	4.80	HR 1.32 (0.91-1.89)
	(US)		Severe : 305	4.3	5.40	HR 1.27 (0.54-3.03)
**Rhee et al. (Current study)**	**Retrospective cohort**	**752,844**	**Mild : 11,438**	**9.6**	**2.48**	**HR 1.10 (0.97-1.24)**
	**(South Korea)**		**Severe : 1,947**	**9.5**	**4.05**	**HR 1.44 (1.14-1.82)**
**Thromboembolic Events**						
Gelfand et al. (2009)	Retrospective cohort	629,412	Mild : 129,143	4.4	3.68	HR 1.06 (1.00-1.10)
	(UK)		Severe : 3,603	3.4	6.05	HR 1.43 (1.10-1.90)
Yang et al. (2011)	Cross-sectional study	6,740	Mild : 1,384	-	-	OR 1.09 (0.66-1.83)

	(Taiwan)		Severe : 301	-	-	OR 1.03 (0.80-1.34)
Ahlehoff et al. (2012)	Retrospective cohort	4,518,484	Mild : 36,765	5.0	4.54	RR 1.25 (1.17-1.34)
	(Denmark)		Severe : 2,793	4.7	6.82	RR 1.65 (1.33-2.05)
Yeung et al. (2013)	Cross-sectional study	95,954	Mild : 4,523	-	-	OR 0.98 (0.71-1.35)
	(UK)		Severe : 1,081	-	-	OR 2.50 (1.46-4.26)
Ahlehoff et al. (2015)[†]	Retrospective cohort	99,357	Mild : 1,693	3.1	4.80	HR 0.99 (0.87-1.11)
	(Denmark)		Severe : 549	2.8	6.10	HR 1.27 (1.02-1.57)
Ogdie et al. (2015)	Retrospective cohort	219,997	Mild : 134,095	5.4	2.50	HR 1.08 (0.99-1.17)
	(UK)		Severe : 4,329	4.3	3.10	HR 1.45 (1.10-1.92)
**Rhee et al. (Current study)**	**Retrospective cohort**	**752,844**	**Mild : 11,438**	**9.6**	**5.13**	**HR 1.04 (0.96-1.13)**
	**(South Korea)**		**Severe : 1,947**	**9.5**	**8.14**	**HR 1.26 (1.07-1.47)**

[*] Incidence rates were calculated per 1000 patient-years.

[†] A prospective cohort study by Ahlehoff et al. (2015) included those with both AF and psoriasis, assessing the additive risk of psoriasis on the prevalent AF patients.

Abbreviations: AF, atrial fibrillation; HR, hazard ratio; OR, odds ratio; RR, risk ratio; TE, thromboembolic events; UK, United Kingdom; US, United States.

III. Supplementary Figures

Supplementary Figure 1. Distribution of Propensity Scores and Balance Assessment after Matching

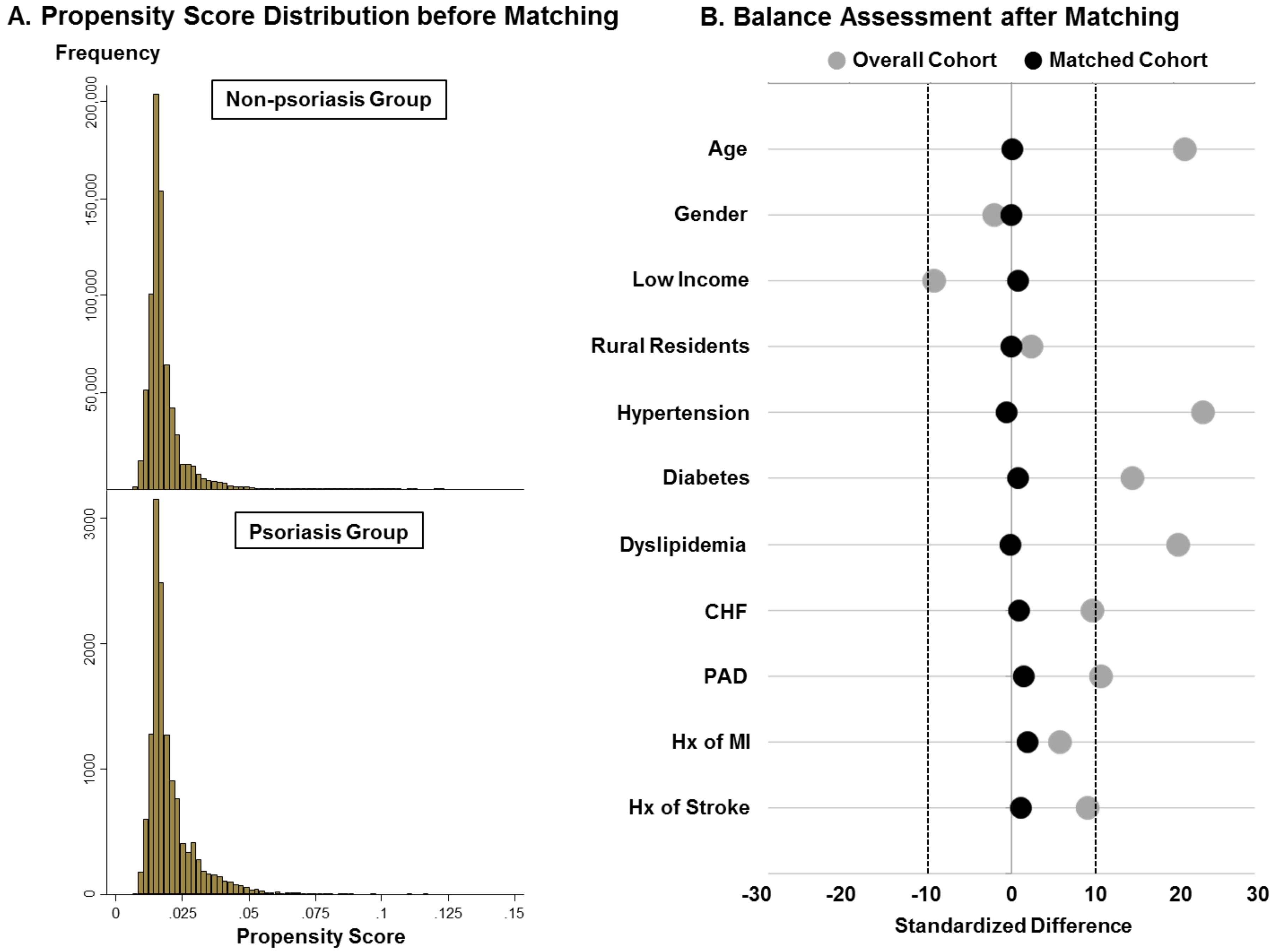

Supplementary Figure 2. Cumulative Incidence of Atrial Fibrillation and Thromboembolic Events in Psoriasis Patients versus Propensity Score Matched Control According to the Severity of Psoriasis

A. Atrial Fibrillation

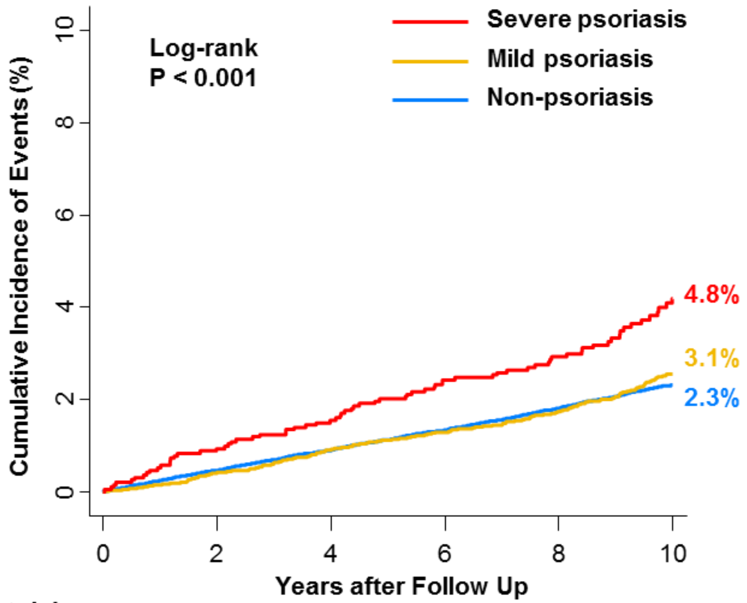

■ Number at risk

Severe psoriasis	1941	1923	1911	1894	1607	964
Mild psoriasis	11416	11369	11313	11268	9244	4700
Non-psoriasis	66785	66477	66188	65901	65584	65250

B. Thromboembolic Events

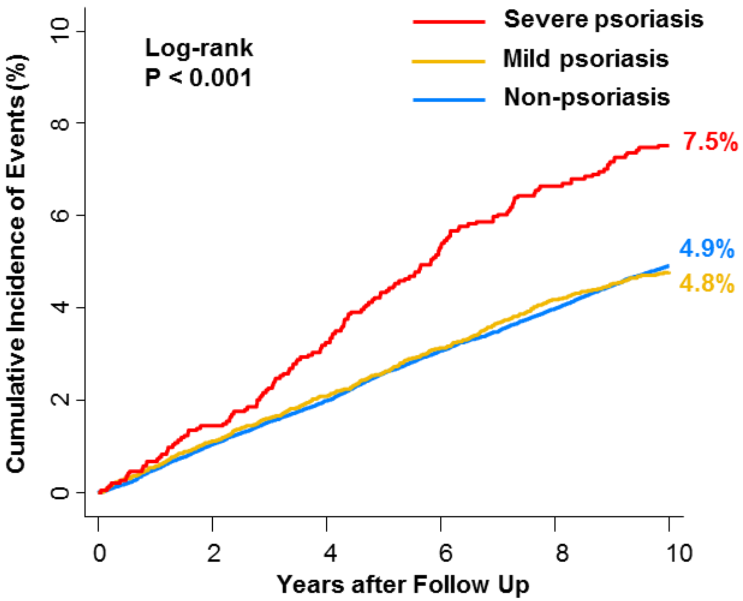

■ Number at risk

Severe psoriasis	1941	1913	1878	1838	1812	1795
Mild psoriasis	11416	11283	11172	11053	10933	10866
Non-psoriasis	66785	66087	65458	64735	64128	63505

Supplementary Figure 3. Subgroup Analyses for Risk of Thromboembolic Events in Psoriasis Patients

Incidence by subgroups (per 1,000 patient-year)	Non-psoriasis (N=739,459)	Psoriasis (N=13,385)	Absolute difference	HR (95% CI)	$P_{interaction}$
**All patients**	**3.63**	**5.56**	**+ 1.93**	**1.53 (1.42-1.65)**	
Male	3.72	5.67	+ 1.95	1.53 (1.38-1.69)	0.922
Female	3.55	5.45	+ 1.90	1.54 (1.38-1.71)	
Age ≥ 65	14.48	17.64	+ 3.16	1.22 (1.10-1.36)	0.007
Age < 65	2.24	3.36	+ 1.12	1.50 (1.35-1.66)	
DM	17.87	19.70	+ 1.83	1.10 (0.94-1.29)	0.002
Without DM	3.13	4.59	+ 1.46	1.47 (1.35-1.59)	
HTN	15.15	17.71	+ 2.56	1.17 (1.06-1.29)	0.374
Without HTN	2.33	2.92	+ 0.59	1.25 (1.12-1.40)	
Dyslipidemia	11.81	14.94	+ 3.13	1.26 (1.10-1.44)	0.234
Without dyslipidemia	3.10	4.33	+ 1.23	1.40 (1.28-1.53)	
CHF	18.22	25.80	+ 7.58	1.41 (1.12-1.76)	0.788
Without CHF	3.46	5.07	+ 1.61	1.47 (1.36-1.59)	
With history of stroke	45.64	54.32	+ 8.68	1.16 (0.96-1.40)	0.072
Without history of stroke	3.31	4.77	+ 1.46	1.44 (1.33-1.56)	
With history of MI	23.80	28.49	+ 4.69	1.19 (0.90-1.57)	0.111
Without history of MI	3.46	5.23	+ 1.77	1.52 (1.40-1.64)	
CHA_2DS_2-VASc score ≥ 2	14.31	17.16	+ 2.85	1.20 (1.09-1.31)	0.262
CHA_2DS_2-VASc score < 2	1.85	2.42	+ 0.57	1.31 (1.16-1.49)	

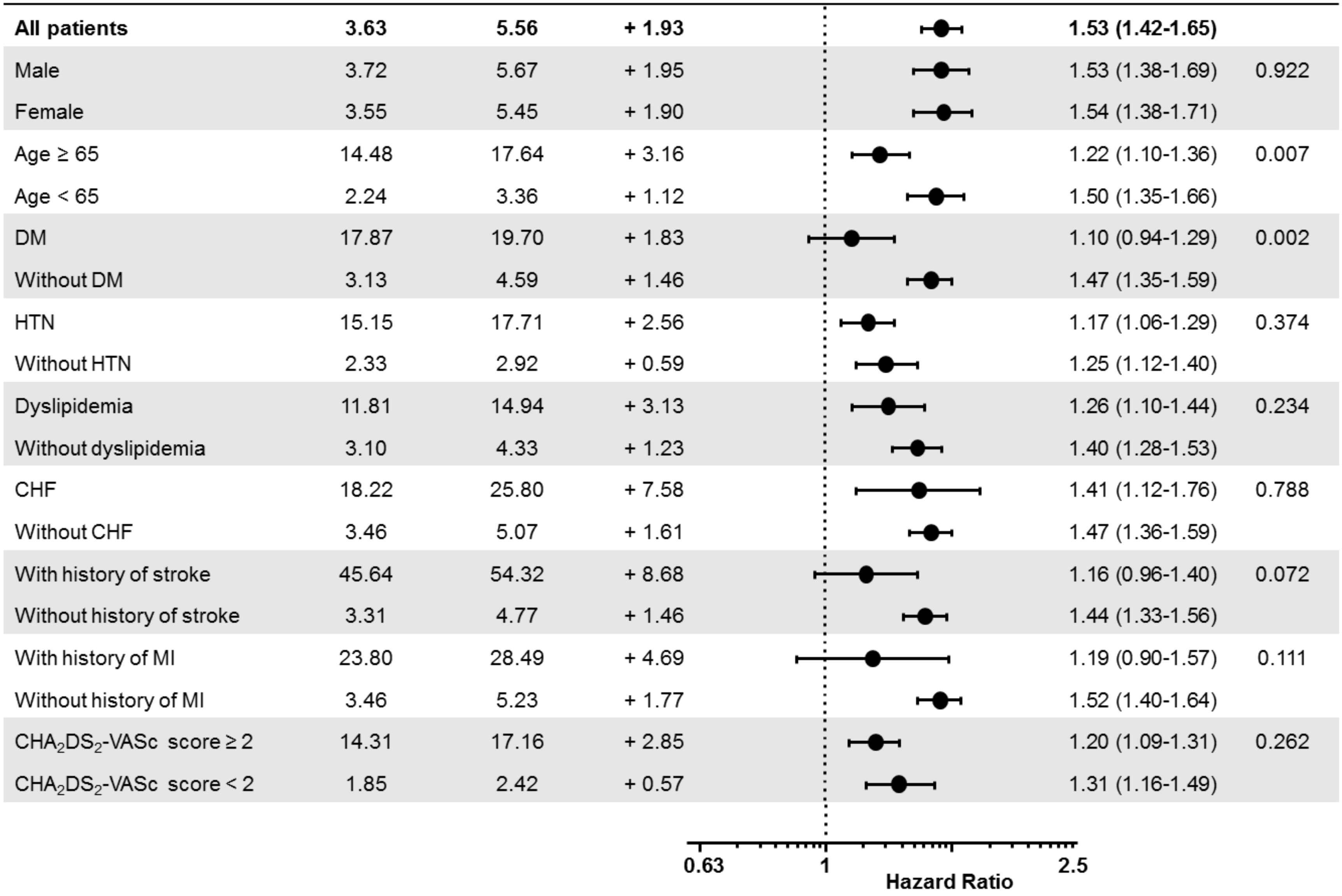

IV. Supplementary Figure legends

Supplementary Figure 1. Distribution of Propensity Scores and Balance Assessment after Matching

(A) The distribution of propensity scores in psoriasis and control groups before matching. **(B)** Standardized differences before and after matching are described, showing successful propensity score matching with standardized differences for all matched covariates being less than 10%.

Abbreviations: CHF, congestive heart failure; Hx, history; MI, myocardial infarction; PAD, peripheral arterial disease.

Supplementary Figure 2. Cumulative Incidence of Atrial Fibrillation and Thromboembolic Events in Psoriasis Patients versus Propensity Score Matched Control According to the Severity of Psoriasis

Kaplan-Meier curves with cumulative hazards of atrial fibrillation **(A)** and thromboembolic events **(B)** derived from propensity score matched cohort compared by severity of psoriasis are presented.

Supplementary Figure 3. Subgroup Analyses for Risk of Thromboembolic Events in Psoriasis Patients

The effects of psoriasis on the risk of TE were shown to be weaker in subgroups with well-known CVD risk factors than those without. However, the absolute risk increment in psoriasis patients who already had CV risk factors.

Abbreviations: CHF, congestive heart failure; CI, confidence interval; CV, cardiovascular;

DM, diabetes mellitus; HR, hazard ratio; HTN, hypertension; MI, myocardial infarction; TE, thromboembolic events.

V. References

1. Egeberg, A. Psoriasis and comorbidities. Epidemiological studies. *Dan Med J*. **63**, (2016).

2. Egeberg, A., Mallbris, L., Gislason, G. H., Skov, L. & Hansen, P. R. Risk of Multiple Sclerosis in Patients with Psoriasis: A Danish Nationwide Cohort Study. *J Invest Dermatol*. (2015).

3. Gelfand, J. M., Neimann, A. L., Shin, D. B., Wang, X., Margolis, D. J., et al. Risk of myocardial infarction in patients with psoriasis. *Jama*. **296**, 1735-1741 (2006).

4. Roh, E., Ko, S. H., Kwon, H. S., Kim, N. H., Kim, J. H., et al. Prevalence and Management of Dyslipidemia in Korea: Korea National Health and Nutrition Examination Survey during 1998 to 2010. *Diabetes Metab J*. **37**, 433-449 (2013).

5. Kim, H. J., Kim, Y., Cho, Y., Jun, B. & Oh, K. W. Trends in the prevalence of major cardiovascular disease risk factors among Korean adults: results from the Korea National Health and Nutrition Examination Survey, 1998-2012. *Int J Cardiol*. **174**, 64-72 (2014).
